# Development of service standards and manpower calculation criteria for hospital clinical pharmacies in South Korea: a survey-based study

**DOI:** 10.1186/s12913-023-10530-7

**Published:** 2024-01-22

**Authors:** Mirinae Lee, Seung-Eun Kim, Jee-Hye Jeong, Yoon-Hee Park, Hye-Won Han

**Affiliations:** Department of Pharmacy, Asan Medical Center, University of Ulsan College of Medicine, 88, Olympic-ro 43-gil, 05535 Seoul, South Korea

**Keywords:** Pharmacy Service, Hospital, Pharmacists, Clinical pharmacy, Workforce, Personnel staffing and scheduling, Surveys and questionnaires

## Abstract

**Background:**

After the revision of the Korean Pharmaceutical Affairs Act, the certification of specialized pharmacists is scheduled to be legally recognized in 2023. Considering that the specialized pharmacist certification was developed based on the working model of hospital clinical pharmacists, it is necessary to establish standards for clinical pharmacists in hospitals and to calculate appropriate manpower. Through this study, we aim to establish practical standards for clinical pharmacists and propose a method for calculating staffing levels based on an investigation of actual workloads.

**Methods:**

This survey-based study consisted of two phases. In the first phase, a literature review was conducted to establish standards for clinical pharmacy services, and tasks in relevant literature were classified to identify clinical pharmacy service tasks that are applicable to the practice of Korean hospitals. Additionally, a preliminary survey was conducted to investigate the essential tasks. In the second phase of the investigation, a multicenter survey was conducted targeting pharmacists in facilities with more than 1,000 beds to explore their perceptions and actual workloads related to tasks.

**Results:**

According to the standards for clinical pharmacists in Korea, clinical pharmacy services consist of a total of 23 tasks, of which 16 have been identified as essential tasks. Essential tasks accounted for 93% of the total tasks in clinical pharmacy services. The average full-time equivalent (FTE) through workload calculation was 2.5 ± 1.9 for each field, while the FTE allocated to actual practice was 2.1 ± 1.6. The distribution of each type of clinical pharmacy service was as follows: 77% for medication therapy management, 13% for medication education, 8% for multidisciplinary team activities, and 3% for medication use evaluation.

**Conclusion:**

This study identified essential tasks common to clinical pharmacy services across different healthcare institutions. However, the FTE of clinical pharmacists in actual practice was insufficient compared to the required amount. In order to establish and expand clinical pharmacy services in a hospital, it is necessary to ensure an adequate workforce for essential tasks.

**Supplementary Information:**

The online version contains supplementary material available at 10.1186/s12913-023-10530-7.

## Introduction

The role of pharmacists is changing due to increases in the complexity of medication treatment, need for multidisciplinary teams, and increases in specialization in the clinical field. Accordingly, pharmacists in South Korea have begun to provide high-quality pharmaceutical services such as clinical pharmacy services [1, 2]. In addition, the role of clinical pharmacists was demonstrated in terms of the reduction of potential prescribing errors, improvement of treatment effects [3–6], and economic evaluation [4, 5]. Clinical pharmacy services are recognized not only in Korea but also worldwide by hospital pharmacists as an essential part of their work, and many are interested in strategies to promote their implementation based on country-specific analyses [7–16].

As of 2022, there are 39,789 pharmacists in South Korea, with approximately 16% (6,426 pharmacists) working in hospitals [17]. According to the 2022 survey report from the Korean Society of Health-System Pharmacists (KSHP), approximately 10% (349 pharmacists) of the total 3,395 pharmacists across 140 hospitals, which averages to about 2.5 pharmacists per hospital, are providing clinical pharmacy services. In 16 tertiary referral centers with more than 1,000 beds, approximately 15% (182 pharmacists) of the total 1,202 pharmacists, or an average of 11 pharmacists per hospital, are providing clinical pharmacy services. Due to a shortage of manpower, there are two main types of clinical pharmacists in South Korea [18]. Clinical pharmacists in multidisciplinary teams can be in charge of specific wards or specific medical departments, while general clinical pharmacists perform specific clinical tasks such as participating in nutrition support teams or therapeutic drug monitoring (TDM) for all wards. In South Korea, as there are no guidelines for operating clinical pharmacists in hospitals and manpower issues, the types of clinical pharmacy operations are different in each hospital.

In other countries, clinical pharmacy services have established standard guidelines and conducted studies to estimate manpower. Japan has a health and welfare system similar to that of South Korea; however, unlike Korea, Japan has a payment compensation system for the medication management of hospitalized patients, which allows clinical pharmacist services to be provided regardless of the number of hospital beds [19]. In Australia, guidelines for the allocation of clinical pharmacists based on the number of patients have been announced [20]. In the United States, the Board of Pharmacy Specialties was established early on, and research was conducted to allocate clinical pharmacists to different clinical settings or pharmacies and determine the required manpower [21–23]. When calculating the clinical pharmacist workforce, it may be beneficial to consider applying guidelines or research results from other countries. However, implementing them directly in Korea could still be somewhat challenging due to differences in systems.

Recently, several studies have been conducted in South Korea to present standards for hospital pharmacist work and measure the workload [24–26]; however, existing research is insufficient to define the standards and scope of clinical pharmacy services and analyze the workload according to detailed work composition. In particular, adequate manpower supply is also linked to work settlement. According to a European study, the UK, which has the highest number of pharmacists per bed, had the highest rate of implementing clinical pharmacy services [27, 28]. Moreover, increasing the number of hospital pharmacists allows them to focus on clinical pharmacy service activities, leading to a reduction in pharmaceutical expenditure [29]. Thus, it is crucial to calculate appropriate clinical pharmacists’ manpower from an economic perspective.

The specialized pharmacist was a private certification introduced by the Korean Society of Health-System Pharmacists (KSHP) based on the clinical pharmacist work of the hospital. KSHP defines specialized pharmacists as “clinical pharmacists who have mastered the field of expertise and have more professional qualities and abilities in medication therapy to contribute to treatment performance and patient health improvement.” After more than 10 years of operation, the Korean Pharmaceutical Affairs Act was recently revised in 2020 and the specialized pharmacist system was legally recognized and is planned to be implemented in 2023 [30]. Starting with the certification of specialized pharmacist, guidelines for an effective operation of clinical pharmacy in South Korea are expected to be needed.

### Aim

This study aimed to investigate and analyze the current status and workload of clinical pharmacists for multiple institutions in South Korea. By reaching an agreement on the essential tasks of clinical pharmacy services, the standard of clinical pharmacy services is presented, and the level of manpower and the degree of manpower demand are calculated through the actual workload. Countries and institutions looking to establish or expand clinical pharmacy services can use the methodology of this study.

## Methods

### Preliminary investigation on clinical pharmacy services at a single center

Through discussions among researchers, we clarified the definition of terms related to clinical pharmacy services that could be included in the survey response guide during the research process by referencing foundational studies [31]. The tasks of clinical pharmacy services were derived from the hospital pharmacist job description published by the KSHP, the association of hospital pharmacists in Korea. To compare the current practices in advanced countries and to identify any omissions or additions, an additional literature review was conducted by examining guidelines published by hospital pharmacist associations in the United States, Europe, the United Kingdom, Australia, and other countries, including the International Pharmaceutical Federation (FIP). Among these, the Society of Hospital Pharmacists of Australia (SHPA) was the only association with independently established guidelines specifically for clinical pharmacy services. Therefore, according to this literature, the tasks were categorized by comparing them with the job description in Korea.

To identify essential tasks, we first conducted a preliminary investigation at a single center before advancing to a two-step process to assess agreement across multiple centers. The investigation focused on clinical pharmacists at Asan Medical Center, which has over 25 years of experience in providing clinical pharmacy services in diverse fields such as pediatrics, critical care, oncology, and organ transplantation. The center serves as a tertiary referral center with more than 2,700 beds. Using Google Docs, the level of agreement on essential tasks was documented using a 4-point Likert scale during a two-round survey (Additional file 2). The threshold for selecting essential tasks was set at an aggregated agreement exceeding 90% across both rounds, calculated as the ratio of positive responses (3 or 4 points) to total responses.

### Calculation of manpower and verification of clinical pharmacy services standards through a multi-center survey 

#### Study design

This study was a survey-based prospective research. A multi-center survey was conducted to analyze the workforce of clinical pharmacists and calculate the manpower demand. According to specialized pharmacist certifications recognized by the KSHP, there are a total of 10 fields in which clinical pharmacists operate. For this study, pediatric pharmacy, organ transplantation pharmacy, oncology pharmacy, and critical care pharmacy, which have been operational in South Korea for several decades, were selected. In general, pediatric pharmacy in Korea targets treatments for critical care, hematology, and oncology, all of which were included in this study.

When recruiting respondents, we tried to reduce the deviation of answers according to the size of the hospital by limiting the target hospitals to those that have more than 1,000 beds. The survey respondents were pharmacists participating in a multidisciplinary team for inpatients. For this study, researchers developed a questionnaire that was refined through a pilot test conducted with pharmacists at Asan Medical Center (Additional file 3). The pilot test was conducted with clinical pharmacists who were not part of the research team. The researchers modified the questionnaire by comparing responses with actual scenarios. With the cooperation of KSHP, the questionnaire was distributed by e-mail to 18 hospitals from August 27 to September 10, 2021. One copy of the questionnaire was requested to be filled out by each clinical pharmacy field, and an answer was requested based on the work from January to December 2020. If there were several pharmacists in a given field, the person in charge of the field responded as a representative. After the collection of the survey results, the respondents were contacted to correct input errors, if necessary.

#### Data collection

Data on the operation status of clinical pharmacy services, perception of each clinical pharmacy service task, and workload were collected with a Microsoft Office Excel-based questionnaire template. The operation status information consisted of the basic characteristics of the hospital, operation information of clinical pharmacists according to the field, and work type information according to the field. The time required per case and frequency were used to calculate the workload of clinical pharmacists. The method used to calculate time and full-time equivalent (FTE), which is derived from a business model, is not commonly applied in pharmacy research. Therefore, we structured our approach by referencing the method proposed in a previous study [32].

i. (Time required for each task) = (Time required per case) $\times$ (Frequency)
ii. (Time required for whole tasks) =
  $$ \sum _{i=1}^{n}\left(\text{T}\text{i}\text{m}\text{e} \text{r}\text{e}\text{q}\text{u}\text{i}\text{r}\text{e}\text{d} \text{p}\text{e}\text{r} \text{c}\text{a}\text{s}\text{e}\right)i\times \left(\text{F}\text{r}\text{e}\text{q}\text{u}\text{e}\text{n}\text{c}\text{y}\right)i$$
  n = total operation.
  i = the individual task.
iii. FTE = Time required for work (A) / working time (B).
  FTE for each task: Apply (i) to (A).
  Total FTE for whole tasks: Apply (ii) to (B).
  (B) is applicable to 8 h, which is the typical one-day working hours of daytime pharmacists.

Perception of each task was investigated to determine whether the essential task and task property were agreed upon. Respondents answered the degree of agreement on the default value for each task on a Likert 4-point scale. We reasoned that the calculation of the required time per case should be judged as an appropriate task, and added task property to the questionnaire. Assuming that the default value of a given clinical pharmacy task (prescription review, intervention, clinical record review and medication history management) was 3 points, respondents were asked to answer the difficulty of other tasks on a 5-point scale. For reference, the criteria of task property were as follows.

- Periodic tasks: tasks that occur frequently and regularly (more than twice a week, usually on a daily basis), but require time calculation for each task through data collection due to expected deviation.
- Specified tasks: tasks that can be performed by specifying the frequency of occurrence and the time required for task, or by assigning a certain amount of time to each hospital.
- Non-specified tasks: tasks that cannot specify the required time due to the large variation in the time required for task regardless of how often the task occurs.

#### Data analysis

The collected data were analyzed using descriptive statistical analysis. Microsoft Office Excel 2016 was used for data processing. The operation status of clinical pharmacies and the workload of clinical pharmacists are presented as mean ± standard deviation or n (%). Median values are presented in addition to the perception of each clinical pharmacy service task. Since the perception of essential tasks and task properties was investigated on a 4-point scale, it was judged that agreements were achieved when the mean and median were 2.5 points or higher. Convergence, consensus, and stability were calculated to identify the degree of agreement by referring to a previous study [33].

## Results

### Preliminary investigation of clinical pharmacy services at a single center

By reviewing the literature, a total of 23 hospital pharmacist tasks being performed in South Korea were selected as clinical pharmacy service tasks. The detailed descriptions of each task outlined in the literature were documented to correspond with real-world practice. Based on their characteristics, clinical pharmacy services were categorized into medication therapy management, medication education, medication use evaluation, and multidisciplinary team activities. For the preliminary two-round survey aimed at identifying essential tasks, 11 clinical pharmacists associated with Asan Medical Center participated. As a result, 16 tasks were identified as essential tasks (Supplemental Table 1).

### Calculation of manpower and verification of clinical pharmacy services standards through a multi-center survey status of multi-center clinical pharmacy services

We sent the questionnaire to 18 hospital pharmacies, of which 9 hospitals responded. Of the hospitals that responded, the average number of permitted beds was 1,669 ± 601, the average number of inpatients per day was 1,346 ± 757, and the average number of registered pharmacist FTEs per daytime on a weekday was 82.0 ± 45.5.

In the field of clinical pharmacy, a total of 26 responses were gathered, which consisted of 8 responses in critical care pharmacy, 7 in oncology pharmacy, 6 in organ transplantation pharmacy, and 5 in pediatric pharmacy. A total of 17 respondents were affiliated with private hospitals, and 9 were affiliated with national or public hospitals. Among the respondents, 62% (n = 16) were affiliated with hospitals where the clinical pharmacy services experience exceeded 10 years. Those with less than 10 years of experience comprised 15% (n = 4) for 5 years or less, 15% (n = 4) for more than 3 years but less than 5 years, and 8% (n = 2) for more than 1 year but less than 3 years.

When calculating the number of pharmacists per patient for the entire field, the average number of patients was 104.0 ± 89.4 and the average number of clinical pharmacist FTEs assigned to actual work was 2.1 ± 1.6. While 16 respondents replied that they reviewed all prescriptions for all patients in each clinical pharmacy field based on the prescription review task, 10 respondents answered that prescription review was performed on patients with a high priority in the multidisciplinary team. A portion of the consultations for TDM, nutrition support, and patient education were conducted by general clinical pharmacists who were not part of the multidisciplinary teams (Table 1).

**Table 1:** Status of multi-center clinical pharmacy services

	Total (N = 26)	Pediatrics (N = 5)	Organ transplantation (N = 6)	Oncology (N = 7)	Critical care (N = 8)
	Mean ± SD (Range)	Mean ± SD (Range)	Mean ± SD (Range)	Mean ± SD (Range)	Mean ± SD (Range)
Patients per day (n)	104.0 ± 89.4 (3.0-316.9)	108.4 ± 16.6 (86.4–128.0)	36.2 ± 28.4 (3.0–80.0)	251.3 ± 69.6 (156.0-316.9)	59.9 ± 32.7 (16.0–99.0)
Total time for clinical pharmacy operation (hr)	16.6 ± 12.5 (0.5–48.0)	23.3 ± 7.3 (12.0-32.5)	6.5 ± 5.2 (0.5–15.4)	19.8 ± 12.8 (8.0–42.0)	17.7 ± 14.7 (4.0–48.0)
Clinical pharmacist manpower allocated to actual practice (FTE)	2.1 ± 1.6 (0.1-6.0)	2.8 ± 0.9 (1.5–4.1)	0.8 ± 0.6 (0.1–1.9)	2.5 ± 1.8 (1.0-5.3)	2.2 ± 1.8 (0.5–60.0)
	**n (%)**	**n (%)**	**n (%)**	**n (%)**	**n (%)**
**Prescription review**					
All patients, all medications	16 (62%)	5 (100%)	3 (50%)	2 (29%)	6 (75%)
Some of priority patients, all medications	7 (27%)	0 (0%)	3 (50%)	2 (29%)	2 (25%)
Some of priority patients, specific medications	3 (12%)	0 (0%)	0 (0%)	3 (43%)	0 (0%)
**Consultation**					
**Therapeutic drug monitoring**					
Clinical pharmacists in multidisciplinary team	15 (58%)	4 (80%)	2 (33%)	2 (29%)	7 (88%)
General clinical pharmacists	7 (27%)	0 (0%)	3 (50%)	4 (57%)	0 (0%)
None	4 (15%)	1 (20%)	1 (17%)	1 (14%)	1 (13%)
**Nutrition support**					
Clinical pharmacists in multidisciplinary team	14 (54%)	5 (100%)	0 (0%)	2 (29%)	7 (88%)
General clinical pharmacists	12 (46%)	0 (0%)	6 (100%)	5 (71%)	1 (13%)
**Medication education**					
**Patient education for specific medication**					
Clinical pharmacists in multidisciplinary team	16 (62%)	4 (80%)	6 (100%)	6 (86%)	0 (0%)
General clinical pharmacists	5 (19%)	0 (0%)	0 (0%)	1 (14%)	4 (50%)
None	5 (19%)	1 (20%)	0 (0%)	0 (0%)	4 (50%)
**Patient education for discharge medication**					
Clinical pharmacists in multidisciplinary team	11 (42%)	4 (80%)	4 (67%)	3 (43%)	0 (0%)
General clinical pharmacists	8 (31%)	1 (20%)	2 (33%)	3 (43%)	2 (25%)
None	7 (27%)	0 (0%)	0 (0%)	1 (14%)	6 (75%)

SD, standard deviation; FTE, full-time equivalent

### Verification of clinical pharmacy services standards through the establishment of essential tasks and perception of each task

Similar to the results obtained at Asan Medical Center, there was agreement on 16 out of 23 total tasks as essential tasks of clinical pharmacy services. In all task property results, 11 periodic tasks, 6 specified tasks, and 6 non-specified tasks were found to have an average score of 3 points or higher, as indicated by the median value. In terms of difficulty, there were 9 tasks with 4 points or more, and all 5 tasks of the multidisciplinary team activity type had a mean difficulty of at least 4 points (Supplemental Table 2).

### Calculation of the workload and manpower demand for clinical pharmacies

One response from the oncology pharmacy field was excluded from data processing under agreement with the respondent because it was not valid, and the workload was calculated as FTE based on 25 responses. While the FTE allocated in actual practice was 2.1 ± 1.6 (Table 1), the manpower of clinical pharmacists calculated based on the entire field was higher at 2.5 ± 1.9 (Table 2). Among the FTE in calculated manpower demand, 2.4 ± 1.8 FTE corresponded to essential tasks, which accounted for 93% of the total calculated manpower demand. The portion of each type of clinical pharmacy service was 77% for medication therapy management, 13% for medication education, 8% for multidisciplinary team activities, and 3% for medication use evaluation (Table 2).

**Table 2:** Distribution of clinical pharmacist manpower demand (full-time equivalent, FTE)

	Total (N = 25)		Pediatrics (N = 5)		Organ transplantation (N = 6)		Oncology (N = 6)		Critical care (N = 8)	
	Mean ± SD (Range)	%	Mean ± SD (Range)	%	Mean ± SD (Range)	%	Mean ± SD (Range)	%	Mean ± SD (Range)	%
**Calculated clinical pharmacist manpower demand**	2.5 ± 1.9 (0.1–6.8)	-	3.5 ± 1.1 (1.8–4.4)	-	0.9 ± 0.7 (0.1–1.9)	-	2.7 ± 1.9 (1.4–5.8)	-	3.0 ± 2.4 (0.6–6.8)	-
**Total, essential tasks**	2.4 ± 1.8 (0.1–6.2)	93%	3.2 ± 1.1 (1.7–4.1)	93%	0.8 ± 0.6 (0.1–1.8)	89%	2.6 ± 1.7 (1.1–5.3)	93%	2.8 ± 2.2 (0.6–6.2)	95%
**Total, periodic tasks**	2.2 ± 1.7 (0.1–5.9)	86%	3.0 ± 1.1(1.6-4.0)	87%	0.7 ± 0.6 (0.1–1.8)	77%	2.4 ± 1.7 (1.0-5.2)	88%	2.5 ± 2.1 (0.4–5.9)	86%
**By task type**										
Medication therapy management	1.9 ± 1.6 (0.0-5.9)	77%	3.1 ± 1.0(1.5–3.7)	89%	0.6 ± 0.6 (0.0-1.6)	59%	1.8 ± 1.1 (0.8–3.5)	66%	2.5 ± 2.0 (0.4–5.9)	85%
Medication education	0.3 ± 0.6 (0.0–3.0)	13%	0.4 ± 0.2(0.1–0.5)	10%	0.3 ± 0.2 (0.0-0.6)	28%	0.7 ± 1.1 (0.2-3.0)	27%	0.1 ± 0.1 (0.0-0.3)	2%
Medication use evaluation	0.0 ± 0.1 (0.0-0.5)	3%	0.1 ± 0.1(0.0-0.3)	3%	0.0 ± 0.0 (0.0–0.0)	2%	0.1 ± 0.0 (0.0-0.1)	3%	0.1 ± 0.2 (0.0-0.5)	3%
Multidisciplinary team activities	0.2 ± 0.2 (0.0-1.1)	8%	0.1 ± 0.1(0.0-0.2)	3%	0.1 ± 0.1 (0.0-0.3)	12%	0.1 ± 0.1 (0.0-0.4)	5%	0.3 ± 0.3 (0.0-1.1)	10%

SD, standard deviation

There were variations in the calculated manpower demand across different clinical pharmacy fields. Pediatric pharmacy showed the highest level of demand for manpower, with an average of 3.5 ± 1.1 FTE. In the field of organ transplantation pharmacy, the workload for medication education was 28%, which was higher compared to other departments, where multidisciplinary team activities accounted for 12%. In oncology pharmacy, the workload for medication education was also high at 27%. On the other hand, critical care pharmacy had a lower workload in medication education at 2% compared to other fields, but the percentage for multidisciplinary team activities was higher at 10% (Table 2).

**Fig. 1:**
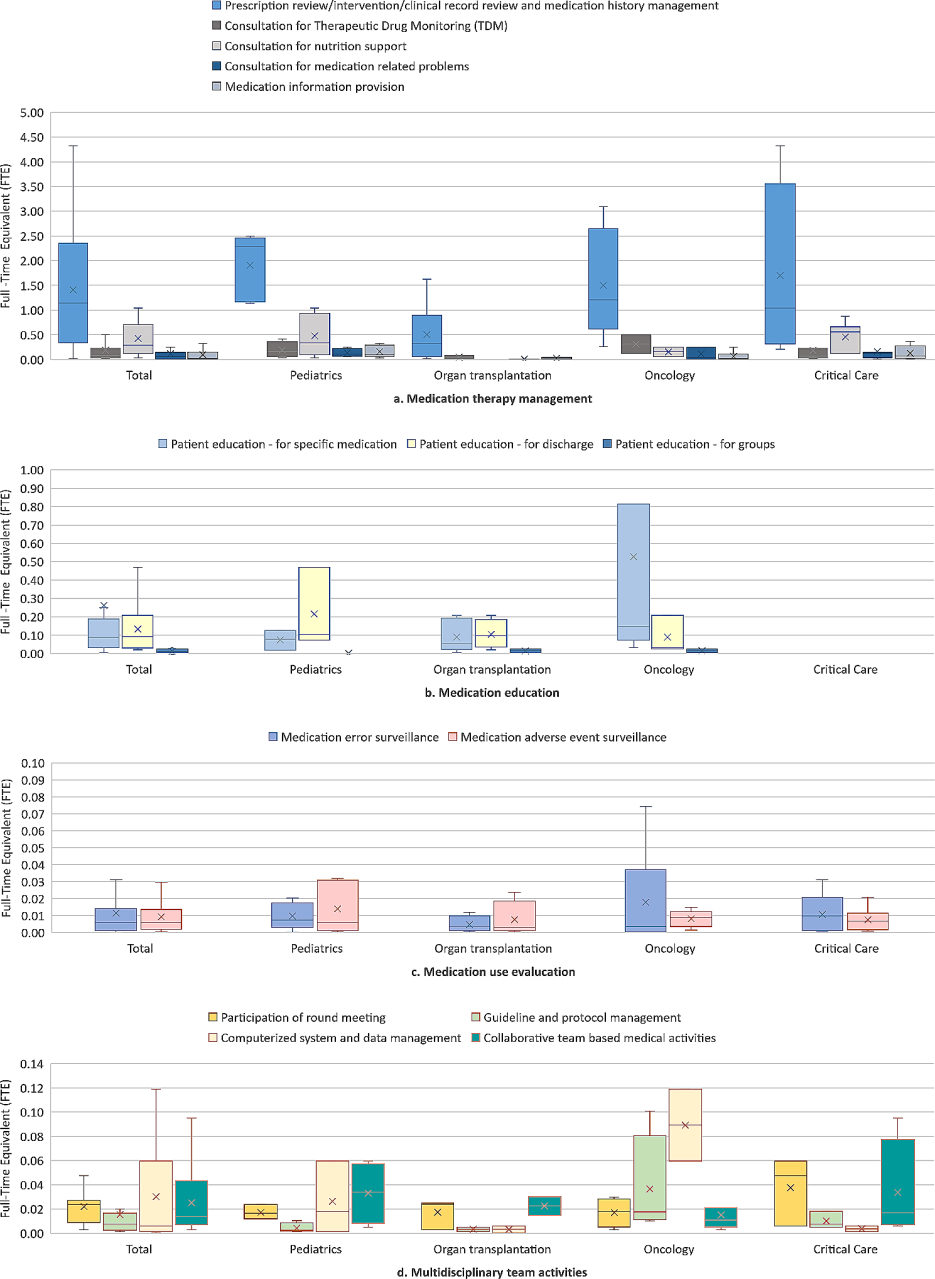
Clinical pharmacist manpower for essential tasks **NOTE**: Due to the high interconnectivity of prescription review, intervention, clinical record review, and medical history management tasks, these were considered as a single task in FTE calculation. The FTE for this task, calculated based on actual workload, was determined to be 1.4 ± 1.2 FTE, showing the highest FTE portion among all fields

Due to the high interconnectivity of prescription review, intervention, clinical record review, and medical history management tasks, these were considered as a single task in the calculation of FTE. The FTE for this task, calculated based on actual workload, was determined to be 1.4 ± 1.2 FTE, showing the highest proportion among all fields (Fig. 1).

In periodic tasks, the total demand for clinical pharmacist personnel was 2.2 ± 1.7 FTE (Table 2). Table 3 shows the time required per case for periodic tasks; a total of 17.1 ± 11.8 min were required for prescription review/intervention/clinical record review/medication history management. Consultation for TDM and nutrition support took 28.0 ± 14.1 min and 29.0 ± 20.7 min, respectively. The time required for medication information provision showed the largest difference by field. The criteria for examples of periodic tasks are shown in Supplemental Table 3.

**Table 3:** Time required per case for tasks with periodic properties

	Total (N = 25)		Pediatrics (N = 5)		Organ transplantation (N = 6)		Oncology (N = 6)		Critical care (N = 8)	
	Time required per case (min)		Time required per case (min)		Time required per case (min)		Time required per case (min)		Time required per case (min)	
	Mean ± SD	Range	Mean ± SD	Range	Mean ± SD	Range	Mean ± SD	Range	Mean ± SD	Range
**Medication therapy management**										
Prescription review/intervention/clinical record review and medication history management	17.1 ± 11.8	(3.0–45.0)	13.0 ± 6.7	(5.0–20.0)	17.5 ± 13.0	(3.0–40.0)	16.2 ± 15.0	(5.0–40.0)	20.0 ± 12.0	(5.0–45.0)
Consultation for TDM	28.0 ± 14.1	(15.0–60.0)	30.0 ± 20.0	(20.0–60.0)	25.0 ± 7.0	(20.0–30.0)	25.0 ± 7.0	(20.0–30.0)	28.6 ± 15.5	(15.0–60.0)
Consultation for nutrition support	29.6 ± 20.7	(15.0–90.0)	30.0 ± 17.9	(15.0–60.0)	N/A	N/A	20.0 ± 0.0	(20.0–20.0)	32.1 ± 26.1	(15.0–90.0)
Consultation for medication related problems	30.8 ± 18.8	(5.0–60.0)	28.8 ± 10.3	(15.0–40.0)	36.7 ± 20.8	(20.0–60.0)	42.0 ± 20.5	(20.0–60.0)	21.4 ± 18.9	(5.0–60.0)
Medication information provision	115 ± 255.7	(5.0-960.0)	45.0 ± 32.0	(5.0–90.0)	210.0 ± 368.9	(30.0-960.0)	36.7 ± 27.3	(10.0–90.0)	146.3 ± 329.1	(10.0-960.0)
**Medication education**										
Patient education – for specific medication	28.6 ± 10.8	(15.0–60.0)	25.0 ± 8.6	(15.0–30.0)	29.2 ± 16.3	(15.0–60.0)	30.0 ± 0.0	(30.0–30.0)	N/A	N/A
Patient education- for discharge	18.3 ± 5.6	(10.0–30.0)	18.3 ± 2.9	(15.0–20.0)	16.3 ± 4.8	(10.0–20.0)	22.5 ± 10.6	(15.0–30.0)	N/A	N/A
**Medication use evaluation**										
Medication error surveillance	20.5 ± 15.9	(5.0–60.0)	15.0 ± 10.6	(5.0–30.0)	31.3 ± 20.1	(15.0–60.0)	25.0 ± 20.0	(5.0–60.0)	12.0 ± 2.7	(10.0–15.0)
Medication adverse event surveillance	22.8 ± 15.7	(5.0–60.0)	27.0 ± 20.1	(5.0–60.0)	31.3 ± 22.5	(5.0–60.0)	18.0 ± 11.5	(5.0–30.0)	17.5 ± 7.6	(10.0–30.0)

SD, standard deviation; TDM, therapeutic drug monitoring; N/A, not available

## Discussion

This study was conducted to establish standards for clinical pharmacy service and to present a standard method of manpower calculation through a multi-center survey. In the clinical pharmacy services standards, we intended to reflect both the theoretical basis based on various literature and the experience-based work status of hospitals in South Korea. In the manpower calculation, we aimed to present the staffing level by reflecting the actual operation status of each hospital.

Previously, a working-level guideline for hospital pharmacies had been proposed; however, there was insufficient data to support clinical pharmacy service standards. According to a recent contract study by the Ministry of Health and Welfare in Korea [34], it was found that among various guidelines, only the SHPA had announced the clinical pharmacy service guidelines. [20]. In South Korea, there is a pharmacist job description provided by the KSHP [35]; however, it does not focus on clinical pharmacy services.

The SHPA guideline suggests 10 activities as clinical pharmacy activities, including medication reconciliation, assessment of current medication management, clinical review, therapeutic drug monitoring, adverse drug reaction management, contributing to the medication management plan, providing medication information, facilitating continuity of medication management on discharge or transfer, participating in interdisciplinary ward rounds and meetings, training and education, participating in research, and quality improvement activities and peer review [20]. Activities such as medication reconciliation and facilitating continuity of medication management, as outlined in the SHPA guidelines, are not classified as separate tasks in our study. Instead, they are included in the tasks of presentation review, intervention, and clinical record review/medication history management. Meanwhile, consultation for nutrition support is included only in our study, not in the SHPA guideline. Many hospital pharmacists in Korea can perform this task because they are guaranteed payment compensation by the healthcare system. Our study is also distinct from SHPA guidelines in that it includes computerized systems and data management. This was done because it is important to manage computerized systems in providing healthcare services in South Korea, given the shortage of medical personnel, including hospital pharmacists.

In South Korea, the number of hospital pharmacists compared to hospital beds is insufficient. In the United States, the pharmacist FTE per 100 beds was 18.6 and the pharmacy technician FTE was 16.2 [36]. In comparison, the number of hospital pharmacists per 100 beds in South Korea was 4.4 in tertiary referral hospitals, 1.74 in general hospitals, and 0.79 in hospitals [34], which significantly differed from the United States. Furthermore, the legal standard for the number of hospital pharmacists in South Korea is based on the volume of prescriptions and is not related to clinical pharmacy services. Currently, health insurance does not typically cover most clinical pharmacy services, with the exception of therapeutic drug monitoring, nutrition support consultation, and certain types of patient education. As a result, healthcare executives are tasked with allocating pharmacists to clinical pharmacy services, and the possibility of reducing the operation cannot be ruled out.

Due to these differences in healthcare environments, it is challenging to directly apply clinical pharmacy service guidelines, especially regarding manpower needs from one country to another. Therefore, we needed to develop guidelines that reflect the Korean situation. However, the clinical pharmacy services standards we developed and our approach to manpower calculation would also be valuable in other countries, especially those where clinical pharmacy services are not firmly established institutionally.

### Strengths and weaknesses

The strengths of this study are as follows. First, we originally developed the concept of essential tasks that must be performed regardless of the hospital’s environment, such as the number of beds or pharmacists. It would be helpful to prioritize tasks in situations where there is insufficient manpower and in institutions where clinical pharmacy services are being launched. Second, by utilizing newly developed Excel-based questionnaire templates, we were able to collect reliable data on workload. In the context of clinical pharmacy services, it is challenging to collect reliable data on workload. In previous studies, there have been attempts to measure the actual time spent on tasks during specific periods [37, 38], or to use the WISN (Workload Indicators of Staffing Need) method developed by the World Health Organization (WHO) [32, 39]. These studies encountered limitations, including challenges in estimating staffing needs for tasks that occur infrequently and difficulties in reaching a consensus on the time required for each task. To address these limitations, we developed a new questionnaire template that automatically calculates FTEs for each task, which allows respondents to estimate the time required per case more accurately, taking into account the proportion of each task. Third, we have newly developed the concept of task properties and classified tasks into those that can be measured by time and those that cannot. In the case of periodic tasks, the time required for each task was also calculated. Measuring the time required for clinical pharmacy services tasks can be challenging, but we could address this issue by introducing the concept of task properties.

The limitations of this study are as follows. First, despite conducting research based on the guidelines and job descriptions provided by the existing hospital pharmacist association, the literature review was limited due to a lack of references. Second, only the workload carried out by clinical pharmacists in the multidisciplinary team was investigated, and the overall workload of clinical pharmacists needed by the entire hospital was not addressed. Third, a survey was conducted on the operation type of clinical pharmacists; however, statistical analysis of the effect of the difference in operation type on the required time was not performed due to the small number of subjects. The main limitation of this study is the small number of respondents, which is due to the lack of manpower in Korea’s clinical pharmacy services, which has led to a scarcity of dedicated clinical pharmacists within multidisciplinary teams. While clinical pharmacy services should ideally be provided in an integrated manner with a patient-centered focus, the current state of clinical pharmacy services is segmented and task-centered. As a result, it was difficult to recruit respondents who met the criteria for our research.

### Statement of key findings

Through this study, an agreement was reached on essential tasks among clinical pharmacy services performed in South Korea. We found that essential tasks accounted for more than 90% of the total workload, and the pharmacist-required FTE calculated based on the actual workload was higher than the pharmacist FTE allocated to work. For tasks that are difficult to establish the required time, the average time per case was calculated through a multi-center survey.

### Interpretation

This study aimed to establish standards and calculate manpower based on actual workload, relying on consensus regarding essential tasks in the absence of clinical pharmacy service guidelines in Korea. Implementing a wide range of tasks at the implementation of clinical pharmacy services can place a significant burden on healthcare management in the early stages. Essential tasks, defined as services that should be provided to patients regardless of the hospital’s environment, are considered the key outcomes of this study. Therefore, we propose that hospitals planning to implement clinical pharmacy services should begin with essential tasks. Additionally, we believe it is reasonable to allocate manpower based on essential tasks and gradually expand operations to implement clinical pharmacy services. The methods used in this study, particularly the selection of essential tasks and the calculation of required manpower, are considered to have significant implications and can be applied as a viable approach in various countries and institutions.

However, the following factors should be taken into consideration. Even if a particular task is deemed essential, the proportion and priority of responsibilities may vary depending on the clinical pharmacy services field. In fact, even within the same type of clinical pharmacy services, the workload varied across different fields in this study. Therefore, it is important to be aware of the specific characteristics of each field and allocate clinical pharmacists according to the proportion of essential tasks in each area. Furthermore, attention should be paid to interpreting and implementing non-specified tasks. While non-specified tasks had little impact on overall manpower demand, they emphasized qualitative aspects such as research, quality improvement activities, and the development of guidelines. Therefore, non-specified tasks should focus on performance-oriented manpower management.

### Further research

This study was based on the current status of hospitals with more than 1,000 beds operating clinical pharmacies in South Korea. Additional studies reflecting this status are needed as clinical pharmacy services spread nationwide in the future.

## Conclusion

As the clinical pharmacy services of hospitals in South Korea differed in the way they operated depending on the institution, standards for work are needed. This study reached an agreement on essential tasks, and it was confirmed that there was a lack of clinical pharmacists operating in preparation for the actual workload. To improve the quality and implementation of clinical pharmacy services, it is necessary to guarantee manpower based on essential tasks. In addition, it is recommended to utilize workload calculation methods, such as time and frequency required for each task, when allocating manpower in countries or hospitals that are planning to implement clinical pharmacy services.

### Electronic supplementary material

Below is the link to the electronic supplementary material.

Supplementary Material 1

Supplementary Material 2

Supplementary Material 3

## Data Availability

The datasets used and/or analyzed during the current available from the corresponding author on reasonable request.
