# Supplementary material for: Development of service standards and manpower calculation criteria for hospital clinical pharmacies in South Korea: a survey-based study
Source: BMC Health Serv Res. 2024 Jan 22;24:118. doi: 10.1186/s12913-023-10530-7 (PMC10802065; doi:10.1186/s12913-023-10530-7)
Supplement: Supplementary file 1 — Supplementary Material 1 [file 12913_2023_10530_MOESM1_ESM.docx]

**Supplemental table 1. Clinical pharmacy service standards for hospital clinical pharmacists in South Korea**

| **Type** | **Task** | **Essential** | **Description** |
| --- | --- | --- | --- |
| **Medication therapy management** | Prescription review | Yes | Review the appropriateness of prescription (e.g., medication selection, treatment duplication, dosage, administration route and method, contraindication and warnings, interaction, allergies, adverse reaction) by determining the characteristics of each patient, each disease, and each medication. |
|  | Intervention | Yes | Plan the medication treatment based on evidence for medication-related problems for each patient, provide recommendation to staff, and record the documentation. |
|  | Clinical record review and medication history management | Yes | Identify and monitor information (e.g., medication history, disease history) in order to continuously manage medication treatment for each patient in charge. |
|  | Consultation for therapeutic drug monitoring | Yes | Assess the effectiveness/toxicity of each patient’s medication concentration using pharmacokinetic knowledge, consult the appropriate dosage, and record the documentation according to the form. |
|  | Consultation for nutrition support | Yes | In consideration of the nutritional status and disease of each patient, identify the problems related to nutrition supply, consult the nutritional requirement or plan, and record the documentation according to the form |
|  | Consultation for medication-related problems | Yes | Evaluate the medication-related problems comprehensively, summarize the evidence-based recommendations, and record the documentation according to the form. |
|  | Medication information provision | Yes | Provide medical staff with up-to-date and accurate medication-related information. Provide answers to medication-related questions from staff. |
| **Medication education** | Patient education for specific medication | Yes | In order to improve medication compliance and understanding of the patient’s own medication, provide education about specific medication or discharge medication for individuals or groups, and record the documentation according to the form. |
|  | Patient education for discharge | Yes |  |
|  | Patient education for groups | Yes |  |
|  | Healthcare team education |  | Provide regular education to the healthcare team for prescribing medication, frequent medication errors, and guidelines for medication use. |
|  | Pharmacist training and education |  | Provide practical education on clinical pharmacy in the field of expertise to pharmacists who are in-hospital or out-hospital, and continue the high-quality practice by enhancing their expertise. |
|  | Student training and education |  | Provide opportunities for career exploration by providing practical training on clinical pharmacy to the college of pharmacy students. |
|  | Extra education provision |  | Provide clinical pharmacy lectures and education in specialized fields at conferences or symposiums. |
| **Medication use evaluation** | Medication error surveillance | Yes | Report and evaluate errors related to medication prescription and administration.  Prepare and apply the method of preventing a recurrence through a systematic review and cause analysis. |
|  | Medication adverse event surveillance | Yes | Report the suspected medication adverse events, identify a causal relationship, and evaluate the possibility of adverse events through literature research. |
|  | Research practice |  | Participate in research based on medication use evaluation and pharmacist interventions. |
|  | Quality improvement |  | Implement improvement activities of process or practice based on medication use evaluation and pharmacist interventions. |
| **Multidisciplinary team activities** | Participation in a round meeting | Yes | Establish and apply a practical medication treatment plan by communicating with patients and staff and observing the actual clinical environment. Increase understanding of the clinical practice site and establish a rapport with staff. |
|  | Participation of conference |  | Share the up-to-date guidelines and medication treatment issues with staff. Apply directly and indirectly to the patient’s medication treatment plan. |
|  | Guideline and protocol management | Yes | Provide evidence-based medication information and manage it in the form of documents/published papers in relation to the publication of the department’s medication treatment guidelines. |
|  | Computerized system and data management | Yes | Manage computerized systems or data according to the guideline related to the department in charge |
|  | Collaborative team-based medical activities | Yes | Communicate with staff on medication-related issues within the team to find effective and practical solutions |

**Supplemental table 2. Perception of essential tasks, task property, and difficulty of clinical pharmacy services**

|  | **Essential** | **Degree of agreement** | | | | | | **Property** | **Degree of agreement** | | | | | | **Difficulty** | |
| --- | --- | --- | --- | --- | --- | --- | --- | --- | --- | --- | --- | --- | --- | --- | --- | --- |
|  |  | **Mean±SD** | **Median** | **Range** | **Convergence** | **Consensus** | **Stability** |  | **Mean±SD** | **Median** | **Range** | **Convergence** | **Consensus** | **Stability** | **Mean±SD** | **Median** |
| **Medication therapy management** | | | | | | | | | | | | | | | | |
| Prescription review  /intervention  /clinical record review and medication history management | Yes | 3.8±0.4 | 4 | (3-4) | 0.00 | 1.00 | 0.10 | Periodic | 3.7±0.5 | 4 | (3-4) | 0.50 | 0.75 | 0.14 | Reference value^a^ | - |
| Consultation for TDM | Yes | 3.5±0.6 | 4 | (2-4) | 0.50 | 0.75 | 0.16 | Periodic | 3.5±0.5 | 4 | (3-4) | 0.50 | 0.75 | 0.14 | 3.7±0.6 | (3-5) |
| Consultation for nutrition support | Yes | 3.5±0.6 | 3.5 | (2-4) | 0.50 | 0.71 | 0.16 | Periodic | 3.6±0.5 | 4 | (3-4) | 0.50 | 0.75 | 0.14 | 3.5±0.5 | (3-4) |
| Consultation for medication-related problems | Yes | 3.6±0.5 | 4 | (3-4) | 0.50 | 0.75 | 0.14 | Periodic | 3.5±0.5 | 3.5 | (3-4) | 0.50 | 0.71 | 0.14 | 4.0±0.7 | (3-5) |
| Medication information provision | Yes | 3.7±0.5 | 4 | (3-4) | 0.50 | 0.75 | 0.13 | Periodic | 3.5±0.7 | 4 | (1-4) | 0.50 | 0.75 | 0.20 | 3.8±0.6 | (2-5) |
| **Medication education** | | | | | | | | | | | | | | | | |
| Patient education for specific medication | Yes | 3.5±0.7 | 4 | (2-4) | 0.50 | 0.75 | 0.18 | Periodic | 3.4±0.6 | 3 | (2-4) | 0.50 | 0.67 | 0.17 | 3.0±0.6 | (2-4) |
| Patient education for discharge | Yes | 3.4±0.7 | 3 | (2-4) | 0.50 | 0.67 | 0.20 | Periodic | 3.4±0.6 | 3 | (2-4) | 0.50 | 0.67 | 0.17 | 2.7±0.6 | (1-4) |
| Patient education for groups | Yes | 3.0±0.8 | 3 | (1-4) | 0.50 | 0.67 | 0.27 | Specified | 3.3±0.6 | 3 | (2-4) | 0.50 | 0.67 | 0.19 | 2.8±0.7 | (1-4) |
| Healthcare team education |  | 2.6±0.8 | 3 | (1-4) | 0.50 | 0.67 | 0.29 | Specified | 3.1±0.6 | 3 | (2-4) | 0.00 | 1.00 | 0.19 | 3.5±1.1 | (1-5) |
| Pharmacist training and education |  | 2.6±0.9 | 3 | (1-4) | 0.50 | 0.67 | 0.33 | Specified | 3.2±0.5 | 3 | (2-4) | 0.00 | 1.00 | 0.14 | 3.4±0.8 | (2-5) |
| Student training and education |  | 2.8±0.8 | 3 | (1-4) | 0.38 | 0.75 | 0.28 | Specified | 3.3±0.5 | 3 | (3-4) | 0.50 | 0.67 | 0.14 | 3.1±0.9 | (1-5) |
| Extra education provision |  | 2.9±0.7 | 3 | (2-4) | 0.38 | 0.75 | 0.22 | Non-specified | 3.4±0.5 | 3 | (3-4) | 0.50 | 0.67 | 0.14 | 4.2±1.0 | (1-5) |
| **Medication use evaluation** | | | | | | | | | | | | | | | | |
| Medication error surveillance | Yes | 3.4±0.5 | 3 | (3-4) | 0.50 | 0.67 | 0.14 | Periodic | 3.3±0.5 | 3 | (2-4) | 0.50 | 0.67 | 0.16 | 3.4±0.8 | (2-5) |
| Medication adverse event surveillance | Yes | 3.4±0.5 | 3 | (3-4) | 0.50 | 0.67 | 0.14 | Periodic | 3.3±0.5 | 3 | (2-4) | 0.50 | 0.67 | 0.16 | 3.1±0.8 | (1-5) |
| Research practice |  | 3.0±0.6 | 3 | (2-4) | 0.00 | 1.00 | 0.20 | Non-specified | 3.5±0.5 | 3.5 | (3-4) | 0.50 | 0.71 | 0.14 | 4.5±0.7 | (3-5) |
| Quality improvement |  | 2.8±0.7 | 3 | (2-4) | 0.50 | 0.67 | 0.23 | Non-specified | 3.6±0.5 | 4 | (3-4) | 0.50 | 0.75 | 0.13 | 4.1±0.7 | (3-5) |
| **Multidisciplinary team activities** | | | | | | | | | | | | | | | | |
| Participation in a round meeting | Yes | 3.5±0.5 | 3 | (3-4) | 0.50 | 0.67 | 0.14 | Specified | 3.4±0.5 | 3 | (3-4) | 0.50 | 0.67 | 0.14 | 4.2±0.7 | (3-5) |
| Participation of conference |  | 2.8±0.7 | 3 | (2-4) | 0.50 | 0.67 | 0.23 | Specified | 3.2±0.5 | 3 | (2-4) | 0.00 | 1.00 | 0.15 | 4.0±0.9 | (2-5) |
| Guideline and protocol management | Yes | 3.3±0.5 | 3 | (3-4) | 0.50 | 0.67 | 0.14 | Non-specified | 3.4±0.5 | 3 | (3-4) | 0.50 | 0.67 | 0.14 | 4.4±0.7 | (3-5) |
| Computerized system and data management | Yes | 3.2±0.6 | 3 | (2-4) | 0.38 | 0.75 | 0.17 | Non-specified | 3.3±0.5 | 3 | (3-4) | 0.50 | 0.67 | 0.14 | 4.1±1.0 | (2-5) |
| Collaborative team-based medical activities | Yes | 3.3±0.5 | 3 | (3-4) | 0.38 | 0.75 | 0.14 | Non-specified | 3.4±0.5 | 3 | (3-4) | 0.50 | 0.67 | 0.14 | 4.2±0.7 | (3-5) |

^a^Based on the score of the prescription review at 3.0, intervention was investigated as having a difficulty level of 3.9±0.6 (range, 3-5) and clinical record and medical history management were investigated as having a difficulty level of 3.3±0.5 (range, 3-4).

TDM, therapeutic drug monitoring.

**Supplemental table 3. Criteria for examples of periodic tasks**

| **Type** | **Task** | **Criteria for an example of periodic task** |
| --- | --- | --- |
| **Mediation therapy management** | Prescription review | ※ These 3 tasks are performed simultaneously, so they are based on each patient  • 1 day prescription for 1 inpatient (20 to 40 medications/day)  • Included: Reviewing prescription or clinical records, developing resolution plans for medication-related problems, providing interventions, monitoring and recordings in a computerized hospital system |
|  | Intervention |  |
|  | Clinical record review and medication history management |  |
|  | Consultation for therapeutic drug monitoring | • Writing a pharmacokinetics analysis report for each therapeutic drug monitoring medication  • Included: Assessing patient medical information and history/medication history, monitoring effectiveness and toxicity, calculating and interpreting pharmacokinetic factors, recommending dosage, and writing a pharmacokinetic analysis report. |
|  | Consultation for nutrition support | • Writing an advisory report on each case of intensive nutrition support  • Included: Assessing patient clinical information/history/nutritional status/supply, planning solutions for nutrition-related problems and treatment, calculating nutrition requirements, writing an advisory report, and monitoring  • Excluded: time required for a nutrition support team round meeting |
|  | Consultation for medication-related problems | • Writing an advisory report on one comprehensive assessment of medication-related problems for each patient  • (Example) Evaluation of medications that estimate the cause of an adverse reaction, advice on medication adjustment due to pharmacokinetic changes/drug interactions, and advice on adjustment for polypharmacy patients.  • Included: Assessing patient clinical information and history/medication history, determining the adequacy of medication/dose selection, monitoring medication use (e.g., adverse reaction, interaction, precaution)/therapeutic effects, and writing an advisory report. |
|  | Medication information provision | • Providing document types for each case of medication information  • (Example) Mechanism of action, medication comparison, and dose conversion information.  • Specifically in the form of documents or e-mails based on the reference  • Excluded: simple information that can be answered immediately |
| **Medication education** | Patient education for specific medication | • Providing one case of special medication therapy education by each patient  • (Example 1) Patients receiving chemotherapy, those who have undergone organ transplantation, or those with chronic diseases (e.g., diabetes, asthma, renal impairment, hepatic impairment, Parkinson's disease)  • (Example 2) Patients with medication such as anticoagulants and inhalers.  • Included: Assessing patient/medication adherence, educating patient for medication therapy/major adverse reactions/cautions, and writing a document |
|  | Patient education for discharge | • Providing one case of discharge medication education by each patient  • Including: Assessing patient/medication adherence, educating patients about medication therapy/major adverse reactions/cautions, and writing a document |
| **Medication use evaluation** | Medication error surveillance | ※ Refers to reporting tasks performed separately from prescription review and intervention (not related to intervention)  • Evaluating and reporting one case of medication-related error  • Included: Assessing the cause and criticality of possible prescription or administration errors that threaten patient safety and recording in a computerized system |
|  | Medication adverse event surveillance | • Evaluating and reporting each medication-related adverse event  • Included: Identifying symptoms, evaluating cause/severity of suspected adverse medication reactions, and recording in a computerized system |
