# Supplementary material for: Development of service standards and manpower calculation criteria for hospital clinical pharmacies in South Korea: a survey-based study
Source: BMC Health Serv Res. 2024 Jan 22;24:118. doi: 10.1186/s12913-023-10530-7 (PMC10802065; doi:10.1186/s12913-023-10530-7)
Supplement: Supplementary file 2 — Supplementary Material 2 [file 12913_2023_10530_MOESM2_ESM.docx]

**Establishing a consensus on the essential tasks of clinical pharmacists in hospitals**

**'Development of Service Standards and Manpower Calculation Criteria for Hospital Clinical Pharmacies in South Korea: a survey-based study’**

| This study, titled "Development of service standards and manpower calculation criteria for hospital clinical pharmacies in South Korea: a survey-based study," will be conducted with the support of the 23rd research grant from the Korea Hospital Pharmaceutical Education & Research Center in 2021. This study is targeted towards users actively engaged in clinical pharmacy services in hospitals, specifically clinical pharmacists at Asan Medical Center in Seoul.  The purpose of this survey is to establish a consensus on the essential tasks of clinical pharmacy services. Although the scope of clinical pharmacy services is diverse, achieving an agreement on essential tasks is essential. Considering the significant variation in clinical pharmacy services among hospitals in Korea, reaching a consensus on essential tasks is expected to contribute to the establishment of standards that should be performed regardless of the medical institution's environment. Furthermore, this study is anticipated to be valuable information for those entering the field of clinical pharmacy services and for healthcare institutions.  We kindly ask for the active cooperation of clinical pharmacists in participating in this survey. |
| --- |

**[SECTION 1] Demographic information of respondents**

1. **Please choose your specific field of clinical pharmacy services.**
   - 1. Clinical care
     2. Oncology
     3. Etc. (_______________)
2. **Please choose the duration of clinical pharmacy services that you support.**
   - 1. Less than 6 months
     2. 6 months or more and less than 1 year
     3. 1 year or more
3. **Please choose the length of time you worked in the hospital.**
   - 1. Less than 1 year
     2. 1 year or more and less than 3 years
     3. 3 years or more and less than 5 years
     4. 5 years or more and less than 7 years
     5. 7 years or more and less than 9 years
     6. 9 years or more
4. **Please write the age of the respondent.**

________________________

**[SECTION 2] Consensus of essential tasks in the clinical pharmacy services**

The scope of tasks for clinical pharmacists at Asan Medical Center in Seoul is outlined below. Please indicate your agreement by selecting 3 (agree) or 4 (strongly agree) for the essential tasks corresponding to the questions below. If you believe a task does not fall under the category of essential tasks, kindly choose 2 (disagree) or 1 (strongly disagree) and provide a brief reason.

| **[Essential tasks in hospital pharmacy services]**  Tasks that have a direct impact on patient care and therefore must be performed as a basic requirement regardless of the number of beds or assigned clinical pharmacists in the hospital. |
| --- |

| **[Tasks of clinical pharmacy services at Asan Medical Center]**   1. Medication therapy management   ∙ Prescription review  ∙ Intervention  ∙ Clinical record review and medication history management  ∙ Consulation for TDM  ∙ Consultation for nutrition support  ∙ Consulation for medication-related problems  ∙ Medication information provision   1. Medication education   ∙ Patient education for specific medications  ∙ Patient education for discharge  ∙ Patient education for groups  ∙ Healthcare team education  ∙ Pharmacist training and education  ∙ Student training and education  ∙ Extra education provision (non-periodic, as needed, for whoever requests)   1. Medication use evaluation   ∙ Medication error surveillance  ∙ Medication adverse event surveillance  ∙ Research practice  ∙ Quality improvement   1. Multidisplinary team activities   ∙ Participation in a round meeting  ∙ Participation of conference  ∙ Guideline and protocol management  ∙ Computerized system and data management  ∙ Collaborative team-based medical activities (communicate with staff on medication-related issues within the team to find effective and practical solutions) |
| --- |

1. **Is ‘prescription review’ an essential task in clinical pharmacy services?**
    Strongly agree (4), agree (3), disagree (2), strongly disagree (1)
   1. Please provide a brief reason if you selected disagree or strongly disagree in the previous question.

• ________________________________________________

1. **Is ‘intervention’ an essential task in clinical pharmacy services?**
    Strongly agree (4), agree (3), disagree (2), strongly disagree (1)
   1. Please provide a brief reason if you selected disagree or strongly disagree in the previous question.

• ________________________________________________

1. **Is ‘clinical record review and medication history management’ an essential task in clinical pharmacy services?**
    Strongly agree (4), agree (3), disagree (2), strongly disagree (1)
   1. Please provide a brief reason if you selected disagree or strongly disagree in the previous question.

• ________________________________________________

1. **Is ‘consultation for therapeutic drug monitoring’ an essential task in clinical pharmacy services?**
    Strongly agree (4), agree (3), disagree (2), strongly disagree (1)
   1. Please provide a brief reason if you selected disagree or strongly disagree in the previous question.

• ________________________________________________

1. **Is ‘consultation for nutrition support’ an essential task in clinical pharmacy services?**
    Strongly agree (4), agree (3), disagree (2), strongly disagree (1)
   1. Please provide a brief reason if you selected disagree or strongly disagree in the previous question.

• ________________________________________________

1. **Is ‘consultation for medication-related problems’ an essential task in clinical pharmacy services?**
    Strongly agree (4), agree (3), disagree (2), strongly disagree (1)
   1. Please provide a brief reason if you selected disagree or strongly disagree in the previous question.

• ________________________________________________

1. **Is ‘patient education for specific medication’ an essential task in clinical pharmacy services?**
    Strongly agree (4), agree (3), disagree (2), strongly disagree (1)
   1. Please provide a brief reason if you selected disagree or strongly disagree in the previous question.

• ________________________________________________

1. **Is ‘patient education for discharge’ an essential task in clinical pharmacy services?**
    Strongly agree (4), agree (3), disagree (2), strongly disagree (1)
   1. Please provide a brief reason if you selected disagree or strongly disagree in the previous question.

• ________________________________________________

1. **Is ‘patient education for groups’ an essential task in clinical pharmacy services?**
    Strongly agree (4), agree (3), disagree (2), strongly disagree (1)
   1. Please provide a brief reason if you selected disagree or strongly disagree in the previous question.

• ________________________________________________

1. **Is ‘healthcare team education’ an essential task in clinical pharmacy services?**
    Strongly agree (4), agree (3), disagree (2), strongly disagree (1)

10-1. Please provide a brief reason if you selected disagree or strongly disagree in the previous question.

• ________________________________________________

1. **Is ‘pharmacist training and education’ an essential task in clinical pharmacy services?**
    Strongly agree (4), agree (3), disagree (2), strongly disagree (1)

11-1. Please provide a brief reason if you selected disagree or strongly disagree in the previous question.

• ________________________________________________

1. **Is ‘healthcare team education’ an essential task in clinical pharmacy services?**
    Strongly agree (4), agree (3), disagree (2), strongly disagree (1)

12-1. Please provide a brief reason if you selected disagree or strongly disagree in the previous question.

• ________________________________________________

1. **Is ‘student training and education’ an essential task in clinical pharmacy services?**
    Strongly agree (4), agree (3), disagree (2), strongly disagree (1)
   1. . Please provide a brief reason if you selected disagree or strongly disagree in the previous question.

• ________________________________________________

1. **Is ‘extra education provision’ an essential task in clinical pharmacy services?**
    Strongly agree (4), agree (3), disagree (2), strongly disagree (1)
   1. Please provide a brief reason if you selected disagree or strongly disagree in the previous question.

• ________________________________________________

1. **Is ‘medication error surveillance’ an essential task in clinical pharmacy services?**
    Strongly agree (4), agree (3), disagree (2), strongly disagree (1)
   1. Please provide a brief reason if you selected disagree or strongly disagree in the previous question.

• ________________________________________________

1. **Is ‘medication adverse event surveillance’ an essential task in clinical pharmacy services?**
    Strongly agree (4), agree (3), disagree (2), strongly disagree (1)
   1. Please provide a brief reason if you selected disagree or strongly disagree in the previous question.

• ________________________________________________

1. **Is ‘research practice’ an essential task in clinical pharmacy services?**
    Strongly agree (4), agree (3), disagree (2), strongly disagree (1)
   1. Please provide a brief reason if you selected disagree or strongly disagree in the previous question.

• ________________________________________________

1. **Is ‘quality improvement’ an essential task in clinical pharmacy services?**
    Strongly agree (4), agree (3), disagree (2), strongly disagree (1)
   1. Please provide a brief reason if you selected disagree or strongly disagree in the previous question.

• ________________________________________________

1. **Is ‘participation in a round meeting’ an essential task in clinical pharmacy services?**
    Strongly agree (4), agree (3), disagree (2), strongly disagree (1)
   1. Please provide a brief reason if you selected disagree or strongly disagree in the previous question.

• ________________________________________________

1. **Is ‘participation of conference’ an essential task in clinical pharmacy services?**
    Strongly agree (4), agree (3), disagree (2), strongly disagree (1)
   1. Please provide a brief reason if you selected disagree or strongly disagree in the previous question.

• ________________________________________________

1. **Is ‘guideline and protocol management’ an essential task in clinical pharmacy services?**
    Strongly agree (4), agree (3), disagree (2), strongly disagree (1)
   1. Please provide a brief reason if you selected disagree or strongly disagree in the previous question.

• ________________________________________________

1. **Is ‘computerized system and data management’ an essential task in clinical pharmacy services?**
    Strongly agree (4), agree (3), disagree (2), strongly disagree (1)
   1. Please provide a brief reason if you selected disagree or strongly disagree in the previous question.

• ________________________________________________

1. **Is ‘collaborative team-based medical activities’ an essential task in clinical pharmacy services?**
    Strongly agree (4), agree (3), disagree (2), strongly disagree (1)
   1. Please provide a brief reason if you selected disagree or strongly disagree in the previous question.

• ________________________________________________
